# Broad-Band Activatable White-Opsin

**DOI:** 10.1371/journal.pone.0136958

**Published:** 2015-09-11

**Authors:** Subrata Batabyal, Gregory Cervenka, Ji Hee Ha, Young-tae Kim, Samarendra Mohanty

**Affiliations:** 1 Biophysics and Physiology Lab, The University of Texas at Arlington, Arlington, TX, United States of America; 2 Department of cell Biology, University of Oklahoma Health Sciences Center, Oklahoma City, OK, United States of America; 3 Department of Bioengineering, The University of Texas at Arlington, Arlington, TX, United States of America; University of Western Australia, AUSTRALIA

## Abstract

Currently, the use of optogenetic sensitization of retinal cells combined with activation/inhibition has the potential to be an alternative to retinal implants that would require electrodes inside every single neuron for high visual resolution. However, clinical translation of optogenetic activation for restoration of vision suffers from the drawback that the narrow spectral sensitivity of an opsin requires active stimulation by a blue laser or a light emitting diode with much higher intensities than ambient light. In order to allow an ambient light-based stimulation paradigm, we report the development of a ‘white-opsin’ that has broad spectral excitability in the visible spectrum. The cells sensitized with white-opsin showed excitability at an order of magnitude higher with white light compared to using only narrow-band light components. Further, cells sensitized with white-opsin produced a photocurrent that was five times higher than Channelrhodopsin-2 under similar photo-excitation conditions. The use of fast white-opsin may allow opsin-sensitized neurons in a degenerated retina to exhibit a higher sensitivity to ambient white light. This property, therefore, significantly lowers the activation threshold in contrast to conventional approaches that use intense narrow-band opsins and light to activate cellular stimulation.

## Introduction

Optogenetic stimulation provides high temporal precision [1–6], minimal invasiveness [7] and cellular specificity by introducing light-activatable ion-channels into cells by genetic targeting. Selective activation of neurons by millisecond (ms)-pulsed blue light has been demonstrated in culture [5], brain slices, as well as in small animals [8–11]. Currently, the use of optogenetic sensitization of retinal cells, combined with active light stimulation, has allowed the possibility of eliminating the requirement of placing electrodes near the retina. This optogenetic activation method is very promising as it only requires light of moderate intensity (~0.1 mW/mm^2^) that can be delivered from a light emitting diode (LED) or laser [1–2]. The present approaches for optogenetic restoration of vision have used either Channelrhodopsin-2 (ChR2) [12–17] or Halorhodopsin (NpHR) [18], both of which have narrow spectral excitation bands (e.g. blue excitation for ChR2 and yellow inhibition for NpHR). However, clinical translation of optogenetic activation for visual restoration suffers from the use of microbial opsins that possess narrow spectral sensitivities and narrow-band active excitation sources. Thus, all attempts for restoring vision by optogenetic stimulation so far have focused on the development and implementation of an array of intense narrow-band light sources (e.g. LED array [19]) to activate opsin-sensitized cells in the retina. This has two major limitations. Firstly, increasing the light intensity to high enough levels to activate chronically narrow-band opsin-expressing cells may substantially damage residual cellular function that might exist in the diseased or impaired retina. Secondly, although optogenetic stimulation allows positioning of the active illumination source outside the eye ball (in contrast to electrical stimulation [20–21]), achieving higher visual resolution using a micro LED array approach is difficult. In contrast, the use of ambient light stimulation (in a passive manner) would mitigate these limitations, in addition to eliminating the discomfort associated with wearing bulky active-stimulation prosthetics.

Here, we report the development of a ‘white-opsin’ that has broad spectral excitability in the visible spectrum. White-opsin-sensitized cells produce a photocurrent that is an order of magnitude higher compared to that generated by ChR2 under similar photo-excitation conditions, without compromising the fast channel kinetics. The use of fast white-opsin may increase photosensitivity to ambient white light for opsin-sensitized neurons in a degenerated retina. Such an effect is predicted to significantly lower neuronal activation thresholds in contrast to conventional approaches that utilize intense, narrow-band light to actively stimulate cells.

## Materials and Methods

### Construction of white-opsin

**Fig 1:**
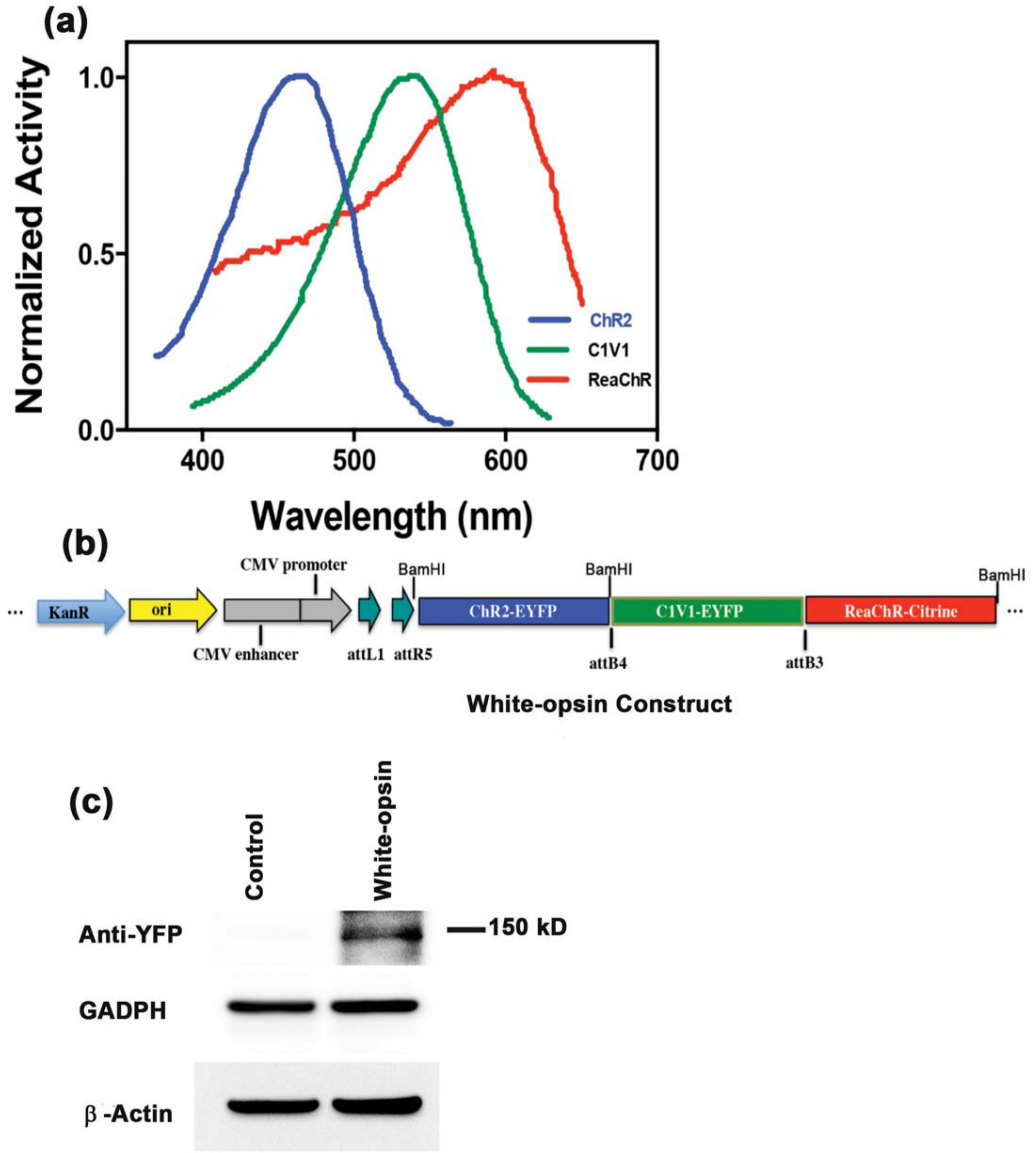
White-opsin construct and broad-band activation. (a) Schematic of normalized activation spectra of narrow-band opsins (ChR2, C1V1 and ReaChR), redrawn from published literature [22–23]. (b) Schematic vector map of the broad-band activatable white-opsin coding region made by fusing three opsin genes of interest encoding ChR2, C1V1 and ReaChR for blue, green and red sensitivity, respectively. The expression of the fusion protein is driven by a CMV promoter. The fusion protein also contains domains that correspond to two different reporter proteins, enhanced yellow fluorescent protein (EYFP) and Citrine. (c) Bands showing control (extracted from HEK293 cells not transfected with white-opsin) and white-opsin (extracted from HEK293 cells transfected with white-opsin).

**Fig 2:**
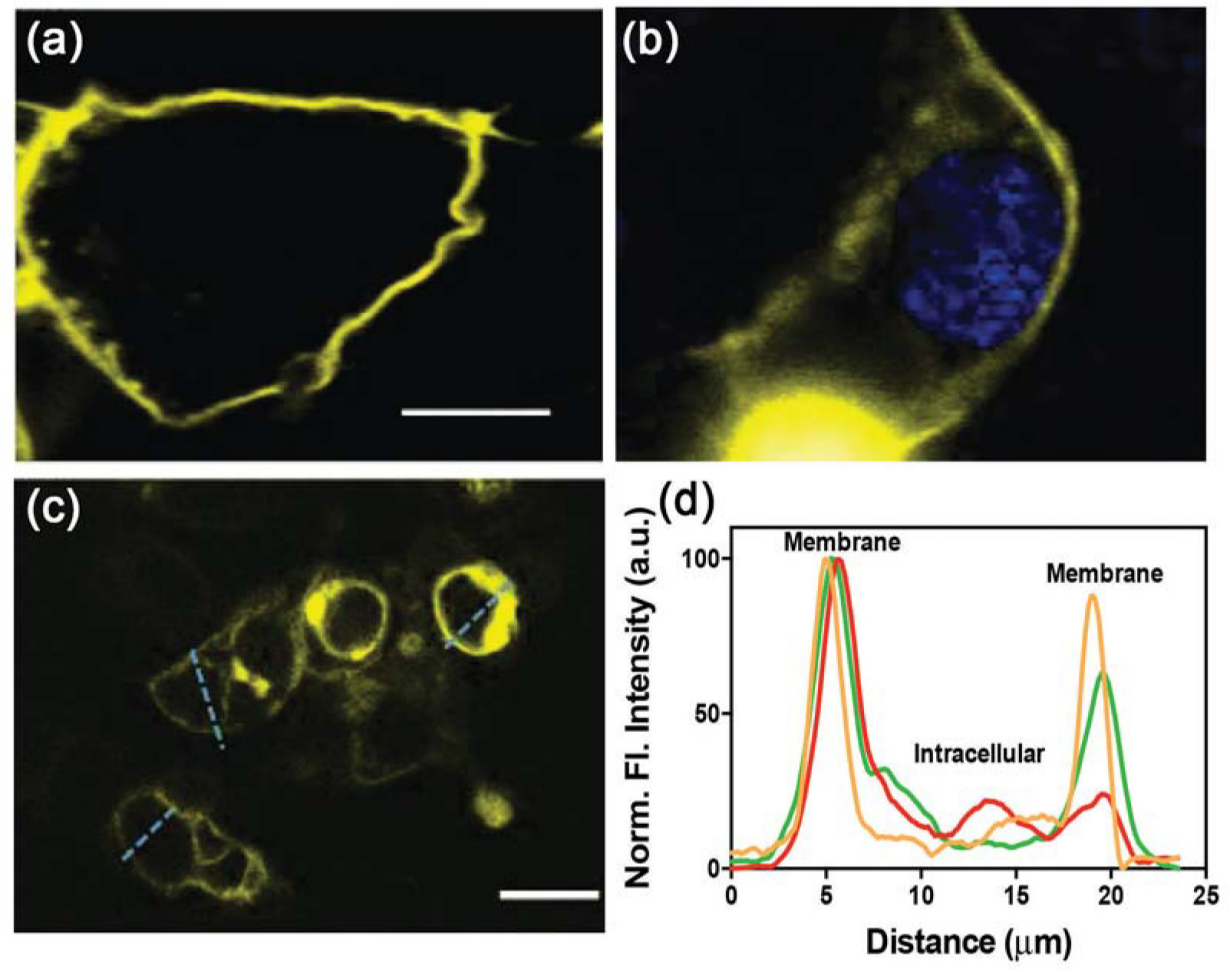
Expression of white-opsin in mammalian cells. (a) Representative confocal image of a white-opsin-transfected HEK293 cell without nuclear staining. Scale bar: 5 μm. (b) Confocal fluorescence image of white-opsin transfected live HEK293 cells stained with nuclear staining dye Hoechst 33242. (c) Confocal image of multiple HEK293 cells showing white-opsin expression. Scale bar: 10 μm. (d) Reporter fluorescence intensity along lines across representative cells (shown in c, dotted blue lines) showing localized expression of white-opsin at the plasma membrane. a.u. = arbitrary units.

The opsins ChR2 (Stanford University), C1V1 (Stanford University) and ReaChR (UCSD) have spectrally-separated photo-activation peaks at 460 nm (blue), 540 nm (green) and 600 nm (red) respectively with a band width of ~90 nm [22–23]. Fig 1A shows the normalized activation spectra of these three narrow-band opsins [23]. By combining all three opsins, the construction of a “white-opsin” with a broad-band activation spectrum that is stimulated by white light should be possible. To test this, MultiSite Gateway® Technology [24] was used to fuse multiple opsin-encoding genes, ChR2, C1V1 and ReaChR, under the control of a cytomegalovirus (CMV) promoter (Figs 1 and 2). A plasmid map of this white-opsin is shown in Fig 1B.

MultiSite Gateway® Technology [25–27], which employs site-specific re-combinational cloning, was used to simultaneously clone multiple DNA fragments in a defined order. Four PCR products, each containing a gene of interest flanked by specific attB or attBr sites, and four donor vectors were used in separate BP recombination reactions to generate four entry clones. The four entry clones and the destination vector, pDEST™ R4-R3 Vector II were used together in a LR recombination reaction to create the white-opsin expression plasmid. Suitable primers were used to remove the stop codon from the respective genes during PCR amplification. Opsin plasmid maps containing ChR2-YFP (Yellow Fluorescent Protein), C1V1-YFP, ReaChR-Citrine, as well as the CMV promoter sequence are shown in S1 Fig. This figure also shows the final expression vector for the white-opsin with the attB sites created by PCR amplification. To ensure the white-opsin fusion-protein was intact and functional, each component (i.e. ChR2, C1V1 and ReaChR) was confirmed by molecular analysis and experimentally by measuring the inward photocurrent generated by activation at particular wavelength.

### Molecular analysis of white-opsin

Specific primers were designed and used to check for sequence fidelity by running a sequencing reaction on a 3130xL Genetic Analyzer, after overnight incubation of the plasmid with the primer. Western blot experiments were performed to confirm expression of the white-opsin protein by using an anti-YFP antibody (Santa Cruz Biotechnology). Loading controls were also included in the western blots by using antibodies to GAPDH (CALBIOCHEM) and β-Actin (Cell Signaling). The molecular weight of the white-opsin (~150 kD) is consistent with that estimated using an open source online tool (http://www.sciencegateway.org/tools/proteinmw.htm).

### Cell culture and opsin transfection

10,000 HEK293 cells (ATCC) were cultured in a 35 mm petridish and maintained in DMEM/F-12 (Life Technologies), supplemented with 10% fetal bovine serum (Sigma-Aldrich), 0.2 mg/mL streptomycin (Sigma-Aldrich) and 200 U/mL penicillin (Sigma-Aldrich). The cultures were maintained at 37°C in a 5% CO_2_ humidified atmosphere. Two days after plating, the cells were transfected with ChR2 or white-opsin constructs (1 μg/ml) using lipofectamine (Life Technologies). After a further 24 hours, reporter protein fluorescence (i.e. Citrine, YFP) was visualized under suitable illumination to identify cells for patch-clamp electrophysiology that expressed the transgene (Fig 2). Prior to light activation, cells were loaded with all-*trans* retinal (ATR; 1 μM, Sigma-Aldrich) for at least 6 hours before conducting downstream experiments. HEK293 cells (without opsin transfection) were cultured separately as negative controls.

### Optogenetic stimulation

**Fig 3:**
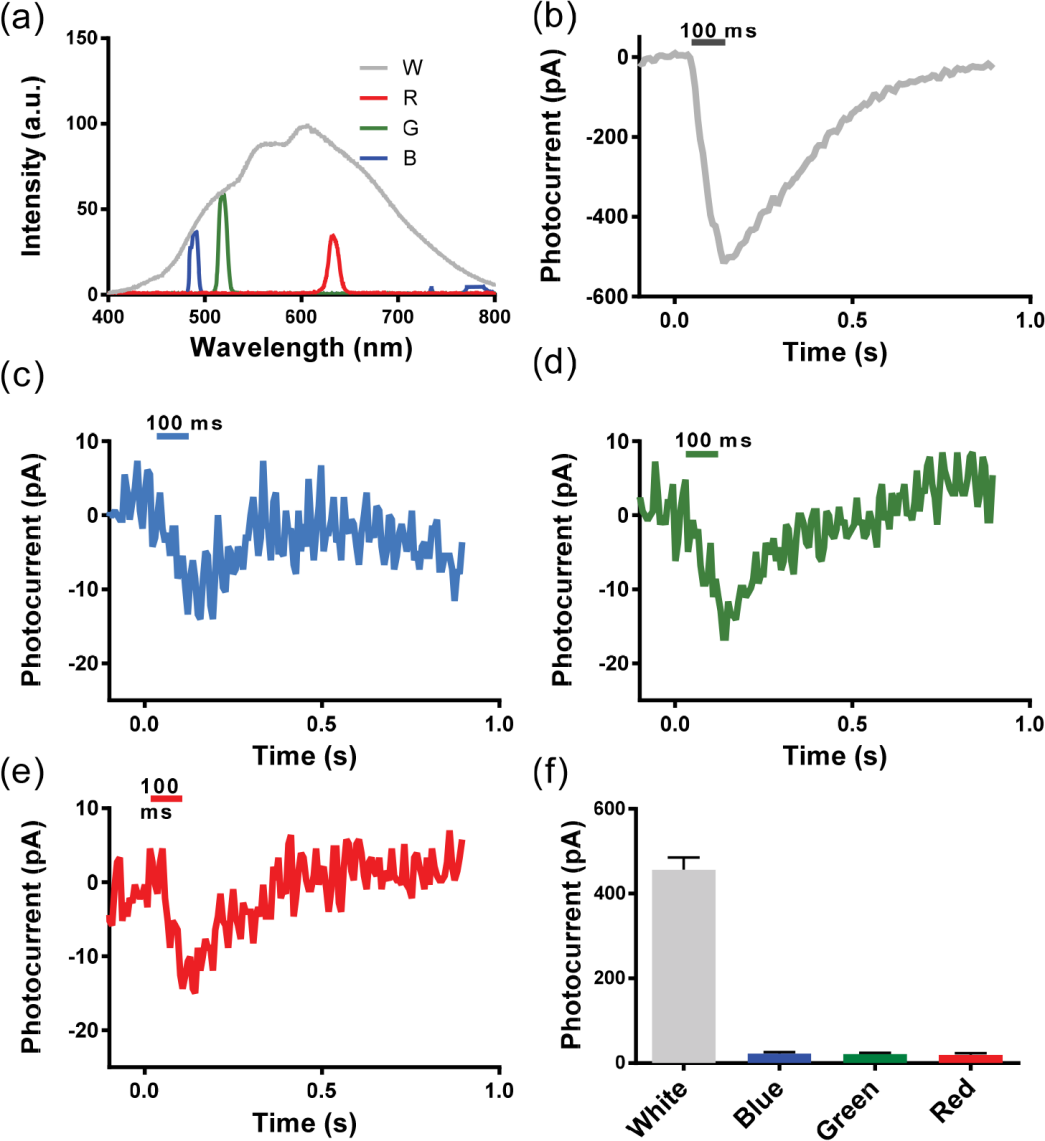
Quantitative comparison between broad-band and spectrally-filtered narrow-band optogenetic activation of white-opsin. (a) Spectra of white-light and spectrally-filtered (not intensity-calibrated) narrow-band lights used for optogenetic activation of white-opsin-expressing cells. Representative inward current responses of white-opsin-sensitized HEK293 cells stimulated by (b) white, (c) blue, (d) green and (e) red light. The white light power is fixed and then spectrally-filtered (lower powers for the narrow band measurements). (f) Measured inward peak-currents for varying spectral excitation of white-opsin-sensitized cells. N = 3 biological replicates (total of five cells, 20 sweeps). Average ± S.D. a.u. = arbitrary units.

Broad-band white light from a 150 W halogen lamp (FO-150, Chiu Technical Corporation) was delivered to the sample by a multimode optical fiber for optogenetic stimulation. The schematic setup for evaluating broad spectral excitation of opsin-expressing cells is shown in S2 Fig. The current-controller was used to regulate the intensity of white light. A set of filters was used in the light path to illuminate the sample with narrow-band blue (490 nm), green (525 nm) and red (630 nm) light. In order to measure the spectrum of the white light and the filtered narrow-band light, a spectrometer (Ocean Optics) was used. As compared to the 300 nm bandwidth of the white light, the spectrally-filtered narrow-band lights had bandwidths of 10 nm (Fig 3A). Note that the narrow bandwidth activation lights used were not positioned at the spectral peak of absorbance (λ_max_) for each of the component opsins; however, they were within the full width at half maximum (FWHM) range of the activation spectrum (Fig 1A). Light power output at the sample plane was measured using a power meter (PM 100D, Thorlabs). For wavelength comparison, the intensity of red light was filtered to the relative intensities of blue and green light using a neutral density filter. The intensity spectral densities (light intensity/nanometer band) of blue, green, red and white light were kept constant at 0.0004 mW/mm^2^/nm. This value corresponds to light intensities of 0.004 mW/mm^2^ for blue, green and red wavelengths (bandwidth: 10 nm), and 0.12 mW/mm^2^ for white light (bandwidth: ~300 nm), respectively. An electro-mechanical shutter controlled the exposure (pulse) duration, which was synchronized by the electrophysiology recording system (Axon Instruments, Molecular Devices). Opsin-expressing cells were exposed to different light conditions (i.e. blue, green, red or white light), where the sequence was randomized. For evaluating the effects of light activation with HEK293 cells expressing the white-opsin, inward currents were recorded by patch-clamp electrophysiological techniques.

### Expression analysis and patch-clamp recording

For analysis of opsin-expression, fluorescence images of reporter fluorescent proteins were acquired using a charge-coupled device (CCD; Photometrics) coupled to an upright fluorescence microscope (Olympus BX 60M). For confirming the localized expression of opsin protein at the cell membrane, confocal microscopy (Zeiss, LSM 510) of cells was carried out in the presence of nuclear staining dye Hoechst H33242.

The opto-electrophysiology set up (S2 Fig) consists of an Olympus upright fluorescence microscope platform with an amplifier system (Axon Multiclamp 700B, Molecular Devices Inc.). Parameters of the pipette puller were optimized in order to obtain desired borosilicate micropipettes of resistance from 3 to 5 MΩ for whole-cell patch-clamp electrophysiology. The micropipette was filled with a solution containing 130 mM K-Gluoconate, 7 mM KCl, 2 mM NaCl, 1 mM MgCl_2_, 0.4 mM EGTA, 10 mM HEPES, 2 mM ATP-Mg, 0.3 mM GTP-Tris and 20 mM sucrose. The electrode was mounted on an XYZ motorized micromanipulator. The standard extracellular solution contained 150 mM NaCl, 10 mM Glucose, 5 mM KCl, 2 mM CaCl_2_, 1 mM MgCl_2_, buffered with 10 mM HEPES (pH 7.3). The output from the amplifier was digitized using Digidata (Molecular devices). For electrophysiological recording, the hardware was interfaced with patch-clamp software (pClamp, Molecular Devices). For the activation of white-opsin-expressing cells, the stimulation beam was delivered by an optical fiber. For generating and controlling pulses of light, the electro-mechanical shutter in the light path was interfaced with a computer. Transistor-transistor logic (TTL) pulses of desired frequency were generated using a Digidata card in order to produce the required pulses of light for activation. Voltage clamp recordings were done in the dark using randomized fluorescent cells derived from three independent experiments. Open-source software (PM MicroManager) and ImageJ (NIH) were used for the acquisition and analysis of fluorescence images, respectively. In case of light stimulation using same wavelength, 100 ms pulse stimulation and ~1 s resting time was given between each sweep of electrophysiological recording in cells. For different color stimulations (blue, green, red or white light), 1 min recovery periods were allowed between each patch-clamp recording. pClamp 10 (Molecular Devices) software was used for all patch-clamp data analyses. Two-tailed Student’s t-test and Bonferroni analyses were used to determine any statistical significant differences in fluorescence and patch-clamp recordings.

### Measurement of activation spectrum

A lens and a grating (600 lp/mm) was used (in place of a narrow-band filter, S2 Fig) to disperse the while light. The grating was rotated to couple light of different wavelengths into the optical fiber (S2 Fig). The expression of each component of the white-opsin fusion protein (i.e. ChR2, C1V1 and ReaChR) was in a 1:1:1 ratio, which was confirmed experimentally by tuning the activation wavelength and measuring the inward photocurrent.

## Results

### Molecular analysis of white-opsin for broad-band light activation

Sequencing data for the white-opsin plasmid confirmed that the whole construct size was ~11 kb, whilst BLAST analysis showed matches to each designed opsin sequence. Western blot analysis confirmed the expression of the white-opsin in HEK293 cells at the expected size of ~150 kD (Fig 1C), whereas no expression was found in negative control lanes despite comparable levels of GAPDH and β-Actin proteins.

### White-opsin is expressed in the cell membrane

The expression and trafficking of white-opsin protein to the cell membrane (compared to the cytoplasm) was quantified by measuring the signal intensity of fluorescent reporters YFP and Citrine. To rule out any contribution from out-of-focus fluorescence (e.g. from cell membranes above and below the plane of focus, which might be considered to be intracellular fluorescence when using an epifluorescence microscope), confocal fluorescence microscopy was carried out. Fig 2 shows that the reporter protein contained within the white-opsin fusion protein is localized to the cell membrane. No detectable intracellular aggregation was observed, implying that trafficking of white-opsin protein to the plasma membrane was efficient. Quantitative comparisons of white-opsin expression in the plasma membrane compared to intracellular areas were carried out by measuring the fluorescence intensity along straight lines that traversed a number of different transfected cells (Fig 2C), where expression levels were shown to be ~6-fold higher at the membrane (Fig 2D).

### Broad-band excitation generates higher photocurrent than narrow-band activation

Fig 3B shows a representative light-induced inward current in a white-opsin-expressing HEK293 cell upon illumination of broad-band white light (pulse width: 100 ms) at a light intensity of 0.12 mW/mm^2^. Representative inward current responses of a white-opsin-sensitized HEK293 cell stimulated by spectrally-filtered blue (490 nm), green (525 nm) and red (630 nm) light stimuli are shown in Fig 3C to 3E. While all narrow-band spectrally-filtered components evoked similar inward currents (~15 pA), white light evoked ~30 times higher inward current (Fig 3F). No statistically significant differences in inward currents were observed between narrow-band blue, green or red experiments. Light stimulation (0.12 mW/mm^2^) did not evoke any inward currents in HEK293 cells without opsin expression (negative control).

**Fig 4:**
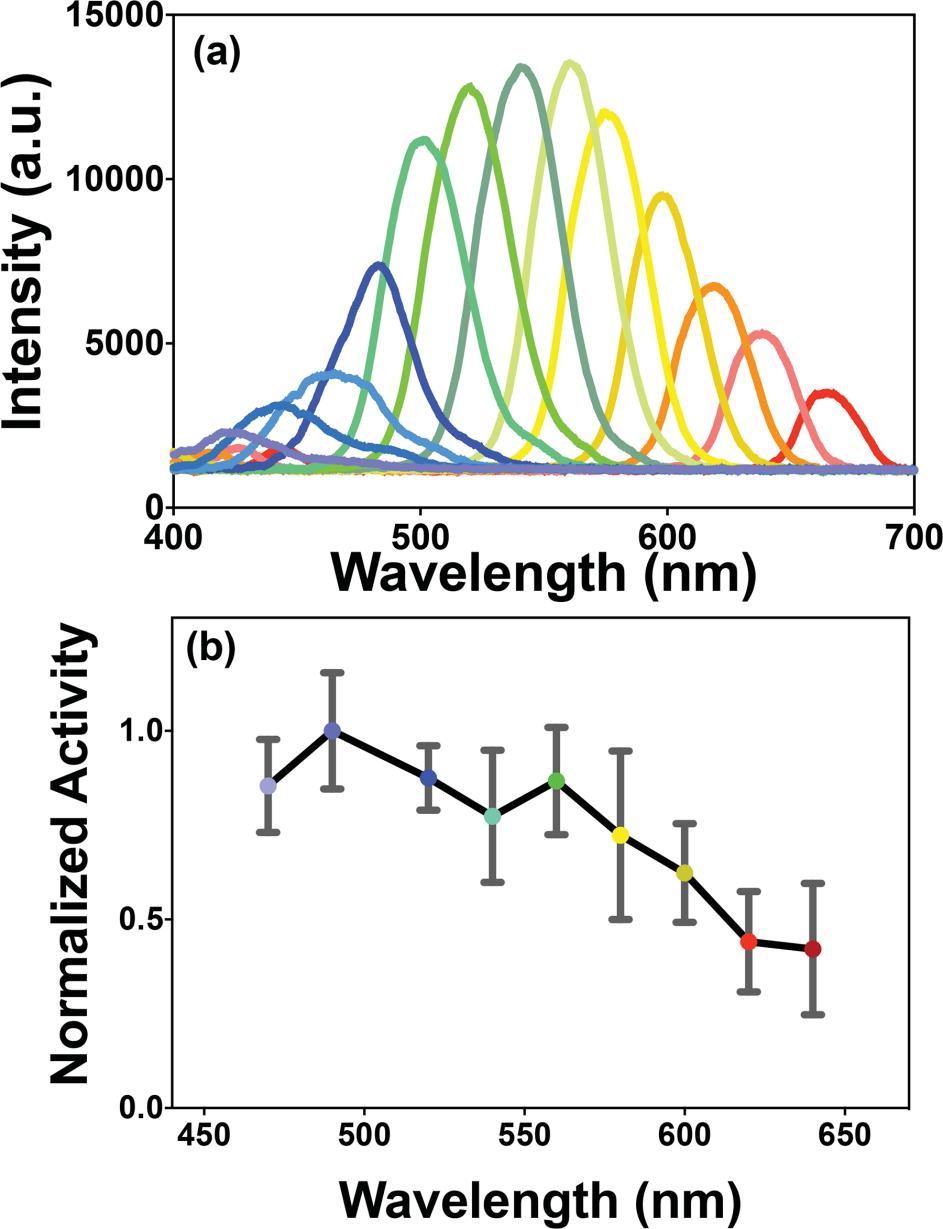
Activation spectrum of white-opsin. (a) Spectra of narrow-band (spectrally-filtered from white light) components measured using Ocean Optics spectrometer. (b) Normalized activation spectrum of white-opsin. Average ± S.D (N = 3 biological replicates, total 25 sweeps per wavelength). This activation spectrum is based on the inward current response of patch-clamped HEK293 cells stimulated with different wavelengths of light. a.u. = arbitrary units.

In order to confirm broad spectral excitability of white-opsin in the visible spectrum, different wavelengths of light ranging from 470 to 640 nm were used, the narrow band spectra of which were measured using an Ocean Optics spectrometer (Fig 4A). Inward photocurrents of white-opsin-expressing cells (N = 3 biological replicates, total 18 sweeps per wavelength) were recorded at different wavelengths of stimulation light and the normalized activation spectrum is shown in Fig 4B. The data show a high level of activity across a 200 nm range of stimulating wavelengths, although the maximal activity level ranged from 50% (at longer wavelengths) to 100% (at shorter wavelengths). The reason behind the differences in the activity levels across the visible spectrum may be attributed to higher efficiency of ChR2 in generating current (upon blue light activation) as compared to ReaChR (upon red light activation).

### White-opsin is excited more by broad-band light than narrow-band opsin

**Fig 5:**
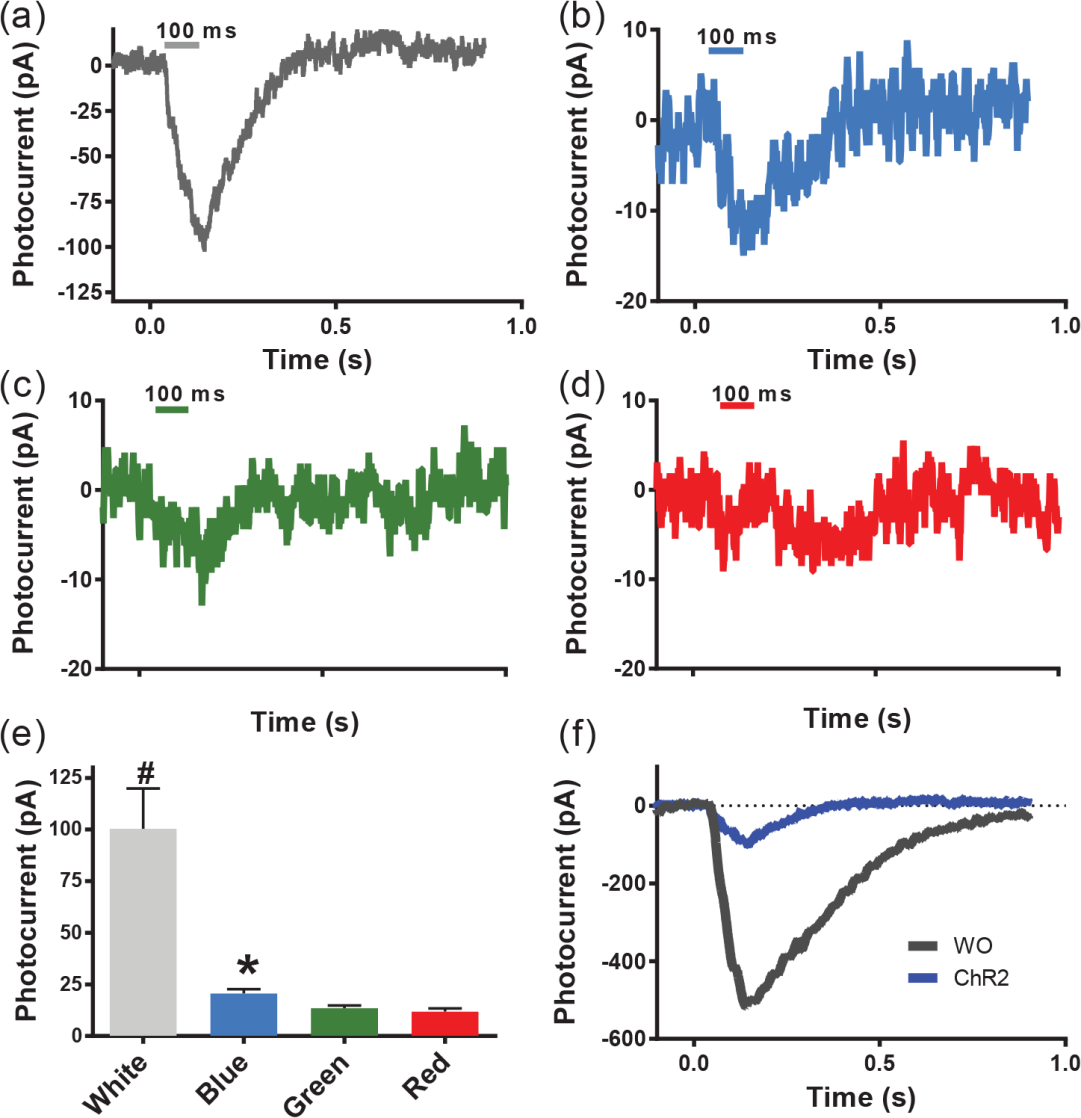
Spectral-dependent activation of narrow-band ChR2-sensitized cells. Representative inward current responses of ChR2-sensitized HEK293 cell stimulated by (a) white light, as well as spectrally-filtered blue (b), green (c) and red (d) components, respectively. The white light power is fixed and then spectrally-filtered (lower powers for the narrow band measurements). (e) Measured inward peak-currents for varying spectral excitation of ChR2-sensitized cells. N = 3 biological replicates (total of 4 cells, 18 sweeps). Average ± S.D. An asterisk (*) represents p<0.05 between blue and other experiments (i.e. green and red). No statistical difference was found between green and red experiments. There is a significant statistical difference between white and other experiments (red, blue and green) (^#^p<0.01). (f) Comparison of representative white light-induced inward currents in a ChR2-expressing HEK293 cell (blue) with a cell expressing white-opsin (gray) at same light intensity. WO: white-opsin.

For evaluating the enhanced light-sensitivity of white-opsin compared to a narrow-band opsin like ChR2, HEK293 cells were transfected with ChR2-YFP only. A representative inward current response of a ChR2-sensitized HEK293 cell stimulated by white light (at 0.12 mW/mm^2^) is shown in Fig 5A. Activation of ChR2-transfected cells by blue, green or red light are shown in Fig 5B–5D. Similar to white-opsin stimulation (Fig 3), the white light power is fixed and subsequently spectrally-filtered (lower powers for the narrow band measurements) to produce differing inward peak-currents in ChR2-sensitized cells (Fig 5E). Compared to an ~15 pA current induced by blue light, green and red light induced small currents of ~5 pA (i.e. the noise level of our instrument). Although white-light stimulation of ChR2 led to a six-fold increase in photocurrent compared to blue light, this is significantly lower than that due to white-opsin (30 times enhancement in light-induced current, Fig 3F). This is because ChR2 has a narrower activation spectrum compared to white-opsin. Further, the inward current evoked by white-light stimulation was found to be significantly lower in ChR2-sensitized cells than that in the white-opsin expressing cells at same light intensity (Fig 5F).

## Discussion

The ultimate goal in using the broad spectrally-sensitive opsin described in this study is to enable ambient white-light (~0.01 mW/mm^2^) to restore vision lost in patients suffering from retinitis pigmentosa (RP) [28–29]. RP and age-related macular degeneration (AMD) are diseases characterized by marked degeneration of retinal photoreceptors, which hinder vision through a lack of light-dependent neuronal activation and a cessation of signal transmission to the visual cortex [29–32]. Cells sensitized with white-opsin show a 30-fold higher photocurrent compared to those produced by using the spectrally-filtered (blue, green, or red) light components only. This 30-fold enhancement in activity can be attributed to the increased bandwidth (~300 nm vs 10 nm) of white-opsin when excited by broad-band white light. Furthermore, cells sensitized with white-opsin show a five-fold increase in inward photocurrent responses compared to cells expressing the narrow-band ChR2 opsin, when stimulated by white light; an increase that can be primarily attributed once again to the broader bandwidth of white-opsin (~300 nm) compared to ChR2 (~90 nm). Although it may be argued that three different plasmids can be delivered separately to encode three different opsins (with spectrally-distinct λ_max_ values), the stoichiometric expression of three spectral-components in each cell cannot be assured as is the case with the fusion protein approach presented here. A limitation may be that the large size of the white-opsin fusion plasmid may hinder its packaging into a single adeno-associated virus (AAV) capsid; however, lentiviral vectors allow for capsid packaging of larger plasmids for delivery to genetically-targeted cells [33]. Also, the use of laser transfection [34] will allow delivery of such large plasmids to specific cells of interest (e.g. photoreceptors or inner retinal neurons).

Use of white-opsin, having broad spectral excitability across the visible spectrum, would facilitate lowering the threshold of white light intensity required to evoke action potentials. This will lead to higher photosensitivity in higher-order neurons sensitized with white-opsin in a degenerated retina under ambient white light conditions. Thus, we believe that the use of white-opsin will minimize light-induced chronic damage to the opsin-expressing cells themselves, as well as the residual light-sensing cells that might exist in a diseased or impaired retina. Although step-function opsins (SFOs) and stable-SFOs provide greater light sensitivity than other opsins (e.g. ChR2, C1V1, ReaChR), their off-time constants are on the order of tens of seconds (and even several minutes) instead of milliseconds [35]. By contrast, white-opsin, while providing higher broad-band sensitivity, has physiologically-relevant on and off rates for maximal retinal function [36].

By utilizing the whole spectrum, we expect that ambient-light can stimulate white-opsin-sensitized retinal ganglion cells (RGCs) to generate action potentials. We believe that such treatment will lead to the restoration of high-resolution vision (as compared to electrical stimulation) by white-light stimulation of white-opsin-sensitized RGCs (or bipolar cells) at ambient light level. Inclusion of the large dendritic fields produced by RGCs will further lower the threshold of ambient light intensity required to generate action potentials in cells expressing white-opsin with white light illumination. This could ultimately eliminate the requirement for prosthetic devices (e.g. camera based LED/microelectrode-arrays providing active and patterned illumination to the retina) for restoration of vision and lead to higher resolution. Furthermore, there is a need to employ a spatially-targeted delivery method [34] to allow expression of the opsin in areas of maximal retinal degeneration (e.g. periphery in RP and macula in AMD). Therefore, spatially-targeted delivery of white-opsin into higher-order retinal cells in photo-degenerated retinal regions might allow the restoration of vision under ambient light conditions. Collectively, this optogenetic tool has the potential to be an important clinical resource and potential treatment for many retinal diseases.

## Supporting Information

S1 Fig Components of white-opsin. Maps of opsin plasmids cloned via PCR to create attB sites for the generation of a “white-opsin” expression vector containing three opsins, ChR2, C1V1, and ReaChR, and the CMV promoter sequence.
(PDF)

S2 Fig Schematic setup for imaging, stimulation and electrophysiology recording. Cells expressing narrow-band opsin or white-opsin activated by narrow/broad-band light. WL: White light source; L: Lens; S1 & 2: Shutter; MO: 40X Microscope objective; FL: Fluorescence excitation source; DM: Dichroic mirror; Ex: Excitation filter; Em: Emission filter.
(PDF)
