# Supplementary figures and images for: Broad-Band Activatable White-Opsin
Source: PLoS One. 2015 Sep 11;10(9):e0136958. doi: 10.1371/journal.pone.0136958 (PMC4567350; doi:10.1371/journal.pone.0136958)

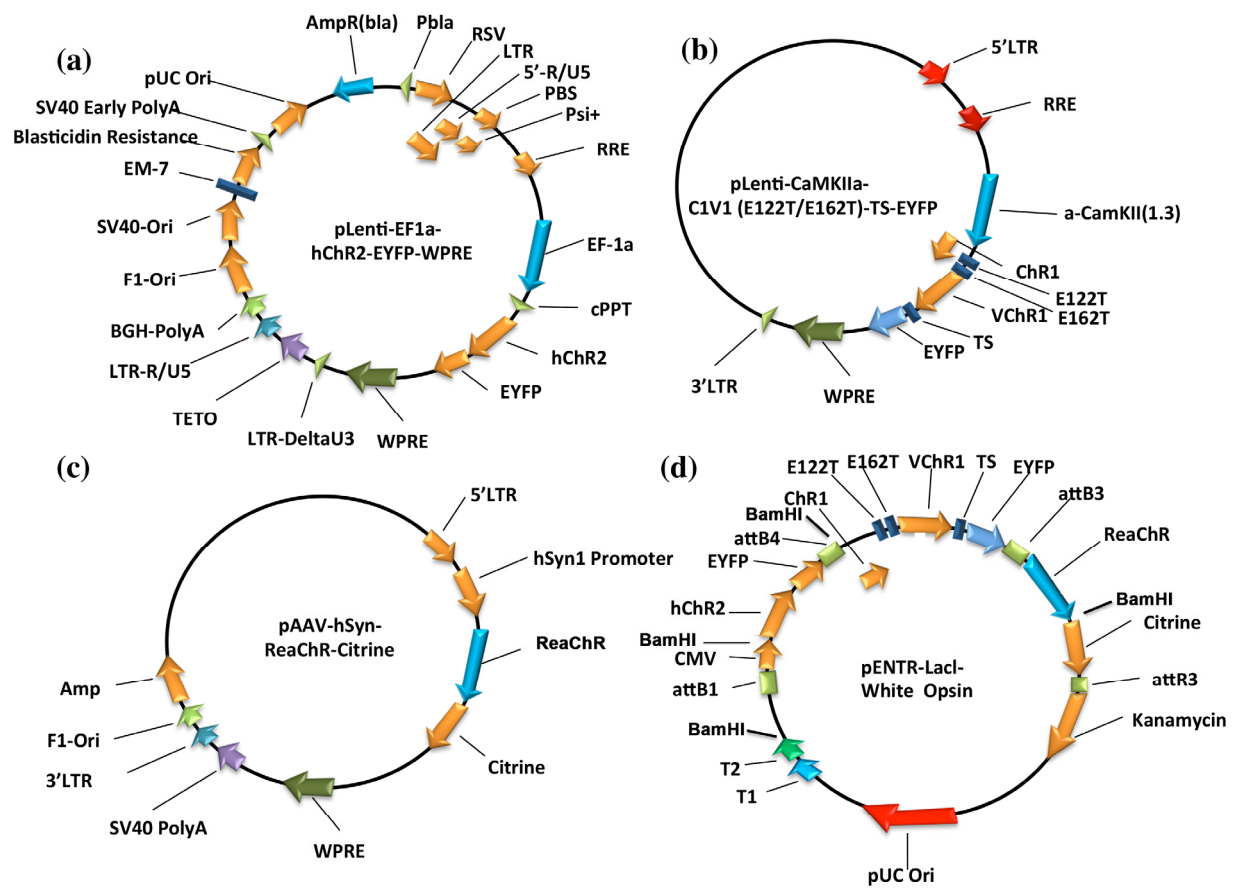

S1 Fig.

Supplement: S1 Fig — Maps of opsin plasmids cloned via PCR to create attB sites for the generation of a “white-opsin” expression vector containing three opsins, ChR2, C1V1, and ReaChR, and the CMV promoter sequence. (PDF) [file pone.0136958.s001.pdf]

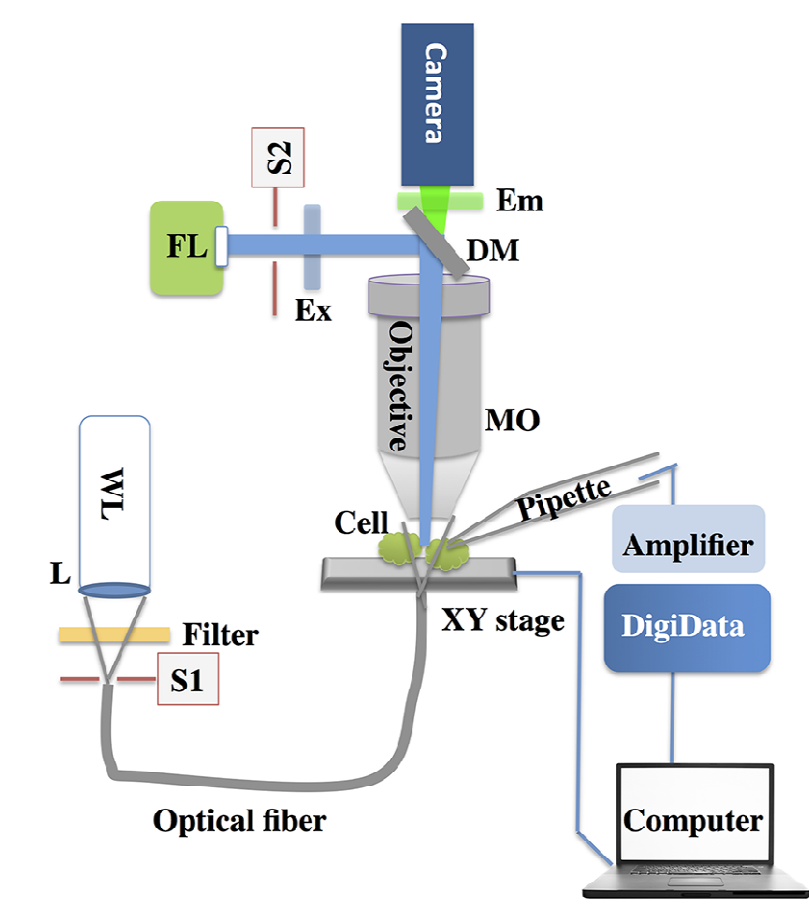

S2 Fig.

Supplement: S2 Fig — Cells expressing narrow-band opsin or white-opsin activated by narrow/broad-band light. WL: White light source; L: Lens; S1 & 2: Shutter; MO: 40X Microscope objective; FL: Fluorescence excitation source; DM: Dichroic mirror; Ex: Excitation filter; Em: Emission filter. (PDF) [file pone.0136958.s002.pdf]
